# Footwear Effects on Postural Stability Are Greater During Unilateral than Bilateral Squat-Based Strength Training Exercises

**DOI:** 10.3390/s26134283

**Published:** 2026-07-06

**Authors:** Stalin Paul Guaigua-Moyolema, Alba Herrero-Molleda, Juan García-López

**Affiliations:** Department of Physical Education and Sport, Faculty of Physical Activity and Sport Sciences, Universidad de León, Campus de Vegazana s/n, 24071 León, Spain; stalinjr1124@gmail.com (S.P.G.-M.); aherm@unileon.es (A.H.-M.)

**Keywords:** biomechanics, postural stability, center of pressure, back squat, split squat

## Abstract

**Highlights:**

**What are the main findings?**

- Conventional sports footwear increased center of pressure displacement and velocity compared with unshod conditions during squat-based strength exercises.
- Footwear effects were greater during the split squat than during the back squat, especially in mediolateral postural control and inter-limb force distribution.

**What are the implications of the main findings?**

- Footwear should be considered when performing, prescribing, or assessing lower-limb strength exercises, especially in tasks with high mediolateral balance demands.
- Footwear selection is important during unilateral exercises, particularly in adults or clinical populations with compromised frontal-plane stability.

**Abstract:**

Footwear may influence postural stability during resistance training, but its effects during bilateral and unilateral squat-based exercises remain poorly understood. This study analyzed the effects of conventional sports footwear on center of pressure (CoP) behavior and vertical ground reaction forces during the barbell back squat and barbell split squat. Eight resistance-trained men completed two sessions. After load determination and familiarization, participants performed both exercises under shod and unshod conditions using a wireless dual force plate system. CoP displacement, CoP velocity, vertical ground reaction forces, and inter-limb vertical force asymmetry were analyzed using a two-way repeated-measures ANOVA. Footwear and exercise type showed moderate or large effects on all analyzed variables, while the footwear × exercise interaction showed moderate or large effects on 12 of 16 variables. CoP displacement and velocity were generally higher under shod than unshod conditions, with moderate to large footwear effects (η^2^_p_ = 0.068–0.772). Mediolateral CoP changes were small in the back squat (0.0–6.2%) but clearly larger in the split squat (18.6–28.8%). During the split squat, the dominant/front limb produced greater force unshod than shod, whereas the non-dominant/rear limb showed the opposite pattern. These preliminary findings from this pilot study indicate that conventional sports footwear can alter postural stability and inter-limb vertical force distribution, especially during unilateral squat-based exercises.

## 1. Introduction

Strength training is a widely used tool for improving physical performance and promoting health, which has led to a marked increase in scientific interest over recent decades [1,2]. From a biomechanical perspective, considerable effort has been devoted to optimizing the technical execution of strength exercises, with the aim of improving their effectiveness and reducing injury risk [3,4,5,6]. In this context, force platforms and stabilometric analysis provide objective information not only on ground reaction forces, but also on postural stability through the assessment of center of pressure (CoP) displacement parameters [7]. This approach may be especially useful in strength exercises, where balance control can influence movement execution, inter-limb load distribution, and the mechanical demands placed on the lower extremities [8].

Among lower-limb strength exercises, the barbell squat is one of the most frequently analyzed from a biomechanical perspective due to its wide application in training, sports performance, and functional rehabilitation [9,10,11]. It is a multi-joint movement whose execution can be modified by different technical factors, such as bar position, foot stance width, trunk inclination, or ankle dorsiflexion, thereby altering load distribution and the mechanical demands placed on the hip, knee, and ankle joints [10,11]. However, squat-based strength training also includes unilateral or asymmetrical variations, such as the split squat, which impose greater balance and unilateral control demands and may modify inter-limb load distribution compared with bilateral tasks. Accordingly, previous research has shown that unilateral and bilateral lower-limb strength exercises differ in joint loading, muscle force distribution, and biomechanical requirements [8,12,13].

In addition to exercise type, footwear is another external factor that may influence balance control during strength exercise execution, since it modifies the interaction between the foot and the support surface [14]. In squat-based exercises, footwear may also alter lower-limb biomechanics through design features such as sole stiffness or heel elevation. Heel elevation, for example, can facilitate squat execution by reducing the ankle dorsiflexion demand required to reach a given depth, promoting greater anterior tibial inclination and a more upright trunk position, and modifying the mechanical demands placed on the lower-limb joints, potentially increasing knee loading while helping to preserve lumbar spine alignment [15,16,17,18]. Consequently, previous research has examined the influence of footwear on back squat biomechanics, mainly focusing on kinematic variables, joint loading, movement performance, and muscle activation [15,19,20,21,22].

Despite this evidence, the influence of footwear on postural stability during resistance exercises remains poorly understood. To the best of our knowledge, the available evidence is currently limited to one deadlift-based study, which reported lower anteroposterior and mediolateral CoP displacement when the deadlift was performed barefoot compared with shod conditions [23]. However, comparable data are lacking for squat-based exercises, and no previous study has examined whether footwear affects postural stability during squat-based exercises, nor whether this effect differs between bilateral and unilateral tasks. This gap is relevant because unilateral exercises may place greater demands on balance control and inter-limb load distribution than bilateral exercises.

Therefore, the aim of the present pilot study was to analyze the effects of conventional sports footwear on postural stability and ground reaction forces during bilateral and unilateral squat-based strength training exercises. It was hypothesized that footwear would increase CoP displacement and velocity, indicating reduced postural stability, and that this effect would be greater during the split squat than during the back squat.

## 2. Materials and Methods

### 2.1. Participants

Eight healthy physically active men participated voluntarily in this study (age: 23.3 ± 2.9 years; body mass: 70.8 ± 4.9 kg; height: 174.1 ± 7.9 cm). All of them had previous experience in resistance training, with a mean training experience of 4.8 ± 2.1 years, a weekly training frequency of 3.5 ± 1.1 sessions, and a mean session duration of 89.4 ± 21.1 min. The main inclusion criteria were having at least 3 years of experience in lower-limb resistance training, performing a minimum of three strength training sessions per week, and being free from lower-limb musculoskeletal injuries related to strength training during the 6 months prior to testing. Prior to data collection, all participants were informed about the study procedures, objectives, benefits, and potential risks, and provided written informed consent. The study was approved by the University Ethics Committee and was conducted in accordance with the Declaration of Helsinki.

### 2.2. Experimental Design

A repeated-measures design was used to examine the effects of footwear and exercise type on force- and balance-related variables during squat-based strength training exercises. The experimental protocol consisted of two sessions separated by 72 h. The first session was designed to familiarize participants with the testing procedures, standardize the foot position and exercise technique, and determine the load corresponding to 10-repetition maximum (10 RM) in both the barbell back squat and the barbell split squat. The second session was devoted to biomechanical testing. Participants performed both exercises under two footwear conditions: shod (i.e., using their habitual conventional sports shoes) and unshod (i.e., wearing their habitual sports socks). During this session, CoP displacement and bilateral ground reaction forces were recorded using a wireless dual force plate system throughout the complete duration of each set. Exercise order and footwear condition were randomized, while exercise tempo, range of motion, and foot position were controlled across conditions. At the beginning of both sessions, participants completed the same standardized warm-up, consisting of joint mobility exercises focused on the lower limbs and one set of 6–8 repetitions with a light load. All sessions were performed in the same sports facility under similar environmental conditions.

**Session 1: Familiarization and Load Determination.** After completing the standardized warm-up described above, participants first performed the barbell back squat. Because all participants were experienced in resistance training, they were asked to select a load close to their habitual 12 RM and then perform the exercise with a load that allowed them to complete 10 repetitions but not an additional repetition. Muscular failure was defined as the inability to maintain the barbell position to complete one more repetition. When necessary, additional attempts were performed, with 5 min of passive recovery between sets. The estimated 1 RM was calculated using the Brzycki equation [24]. According to this equation, the 10 RM load corresponds to approximately 75% of 1 RM.

**Figure 1:**
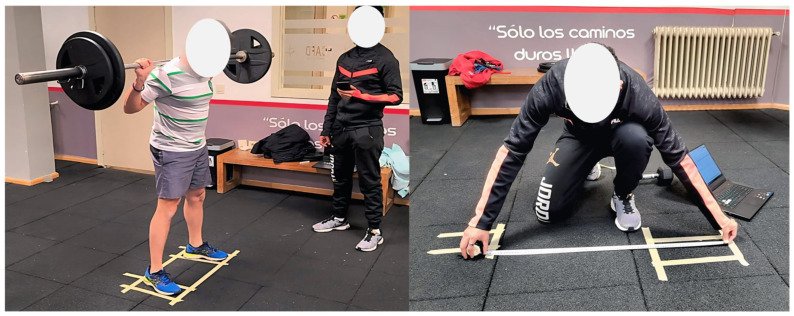
Execution of the barbell back squat during the first session (**left**) and standardization of foot placement for the barbell split squat using tape markings (**right**).

After a 5 min passive recovery period, the same procedure was repeated for the barbell split squat (Figure 1). Participants performed the split squat with their preferred lead leg and were instructed to reach approximately 90° of knee flexion in the front leg at the bottom position, corresponding to the transition from the eccentric to the concentric phase [8]. Similarly, barbell back squat depth was standardized at approximately 90° of knee flexion, defined as the angle between the femur and tibia [25]. In both exercises, range of motion was controlled by visual inspection, with participants instructed to reach the standardized depth in each repetition. They were allowed to choose the most comfortable starting position for each exercise, and foot placement was recorded in both the mediolateral and anteroposterior directions in order to reproduce the same position during the second session. All repetitions were performed using a controlled tempo of 2 s during the descending phase and 1 s during the ascending phase, with no pause at the bottom position (2:0:1 tempo), which was regulated by a metronome set at 60 beats·min^−1^ [25].

**Figure 2:**
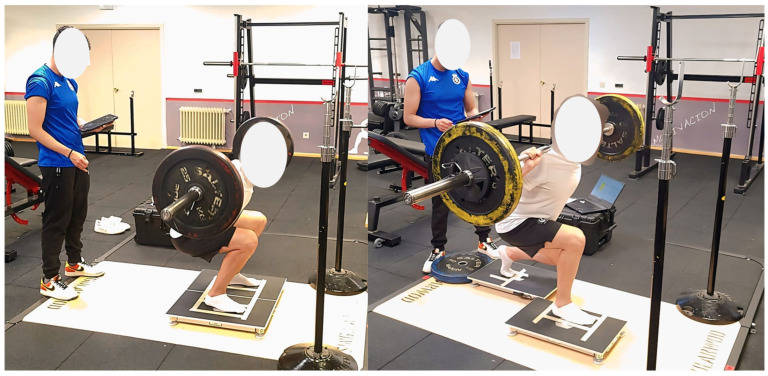
Biomechanical testing during the barbell back squat (**left**) and barbell split squat (**right**) performed on the wireless dual force plate system.

**Session 2: Biomechanical Testing.** After completing the standardized warm-up described above, participants performed the barbell back squat and barbell split squat on the force plates using the 10 RM load determined during the first session (Figure 2). Foot placement was reproduced according to the mediolateral and anteroposterior distances recorded during the first session, and the same exercise technique and movement tempo were maintained across conditions. For each exercise, the same mediolateral and anteroposterior foot positions were reproduced in the shod and unshod conditions using the recorded distances and floor markings. Each participant completed 2 sets of 5 repetitions in each footwear condition for each exercise, resulting in 4 sets for the barbell back squat and 4 sets for the barbell split squat. The order of the exercises and the footwear conditions were randomized. A passive recovery period of 3 min was allowed between sets of the same exercise, and a 5 min passive recovery period was provided between the two exercises. During the barbell split squat, participants used their preferred lead leg, and the same foot placement was maintained across footwear conditions. For the primary analysis (Supplementary Materials), the mean of the two sets performed under each condition was taken as the representative value for each participant [26].

**Instrumentation and Data Collection.** Biomechanical data were recorded during the entire duration of each set using a wireless dual force plate system (Hawkin Dynamics Wireless Dual Force Plates, Hawkin Dynamics Inc., Westbrook, ME, USA), which consisted of two independent uniaxial portable platforms (60.0 × 36.0 × 7.0 cm) that allowed for simultaneous bilateral assessment. The beginning of each recording was determined by the examiner’s start signal, whereas the end of the recording was established when the final repetition was completed. Each platform was equipped with four strain-gauge load cells located at the corners of the plate, providing a reported precision of 0.1 N and recording vertical force data at a sampling frequency of up to 1000 Hz. According to the manufacturer’s description, CoP location is derived from the vertical force data recorded by the four corner load cells of each platform, allowing two-dimensional CoP displacement to be obtained in the anteroposterior and mediolateral directions (https://www.hawkindynamics.com/blog/cop, accessed on 11 May 2026). Previous research has supported the validity of this system for force assessment when compared with laboratory-grade platforms [27,28]. The force plates were connected to a tablet (Xiaomi Pad 5, Xiaomi Inc., Beijing, China) running the manufacturer’s software (Hawkin Capture, version 9.7.0, Hawkin Dynamics Inc., Westbrook, ME, USA), and all records were simultaneously stored in the Hawkin Dynamics Cloud Platform.

For the analysis, the dominant (D) limb was defined as the limb selected by each participant as the lead leg during the barbell split squat. The contralateral limb was defined as the non-dominant (ND) limb. This same classification was maintained for the barbell back squat to allow for comparisons between exercises. The analyzed variables were total vertical ground reaction force, vertical ground reaction force for the dominant (D) and non-dominant (ND) limbs, and inter-limb vertical force asymmetry. The latter was calculated as the absolute difference between the D and ND limbs, divided by the mean value of both limbs and multiplied by 100. Center of pressure (CoP) variables were also obtained separately for the D and ND limbs from each force plate, including mediolateral (ML) and anteroposterior (AP) CoP displacement, CoP sway length, ML and AP CoP velocity, and CoP sway velocity. No combined bilateral CoP variable was calculated. For each condition, variables were calculated from the complete duration of the set, and the mean value of the two sets was used as the representative value for each participant. Vertical GRF variables were expressed as time-average force values over the complete set. ML and AP CoP displacement corresponded to the cumulative distance traveled by the CoP along each respective axis, whereas CoP sway length represented the cumulative two-dimensional length of the CoP trajectory. ML and AP CoP velocity, as well as CoP sway velocity, were calculated point by point from consecutive CoP samples and then the average over the complete duration of the set was computed.

### 2.3. Statistical Analysis

The results are expressed as mean ± SD. Statistical analyses were conducted using SPSS statistical software (v. 26.0, IBM Corp., Armonk, NY, USA). Normality of the within-subject contrasts was assessed using the Shapiro–Wilk test. A two-way repeated-measures analysis of variance (ANOVA) was used to examine the effects of footwear condition (shod vs. unshod), exercise type (barbell back squat vs. barbell split squat), and their interaction on the analyzed force- and balance-related variables. When significant main effects or interactions were observed, post hoc pairwise comparisons were performed using paired-samples *t*-tests with Bonferroni correction. Effect size magnitude was evaluated using partial eta squared (η^2^_p_), and interpreted as small (0.01–0.059), moderate (0.06–0.137), and large (>0.137). Partial eta squared (η^2^_p_) was selected because it is a standard and widely reported effect size measure for repeated-measures ANOVA, allowing for comparison with previous studies using similar statistical designs. Observed statistical power was also calculated for each ANOVA effect. This variable ranges from 0 to 1 and indicates the probability of detecting an effect of the observed magnitude at α = 0.05, with values ≥ 0.80 generally considered adequate according to Cohen’s convention [29]. The statistical significance level was set at α = 0.05.

## 3. Results

Table 1 shows that footwear and exercise type had a moderate or large effect on all analyzed variables (16 out of 16), while the footwear × exercise interaction showed a moderate or large effect on 12 of the 16 analyzed variables. However, observed statistical power exceeded 0.80 in 7 of 16 variables for the footwear effect, 12 of 16 variables for the exercise effect, and 3 of 16 variables for the footwear × exercise interaction.

For the CoP variables, both displacement and velocity were generally higher under shod than unshod conditions (η^2^_p_ = 0.068–0.772), with values generally higher in the split squat than in the back squat (η^2^_p_ = 0.234–0.861). The footwear × exercise interaction was more pronounced for mediolateral CoP variables (η^2^_p_ = 0.444–0.582) than for anteroposterior variables (η^2^_p_ = 0.052–0.125). To facilitate practical interpretation, the main footwear-related differences in mediolateral CoP variables were also expressed as relative changes, showing small increases in the back squat (0.0–6.2%) and larger increases in the split squat (18.6–28.8%).

**Figure 3:**
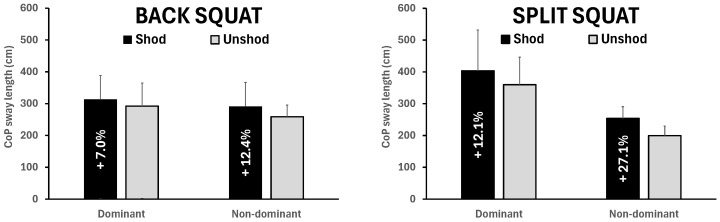
Center of pressure (CoP) sway length for the dominant and non-dominant limbs during the back squat (**left**) and split squat (**right**) under shod and unshod conditions. Values are expressed as mean ± SD. Values inside the bars indicate the relative increase under shod compared with unshod conditions.

Figure 3 shows that CoP sway length was greater under shod than unshod conditions in both limbs and exercises. Footwear-related differences were larger in the split squat than in the back squat for both the dominant limb (12.1% vs. 7.0%) and the non-dominant limb (27.1% vs. 12.4%).

**Figure 4:**
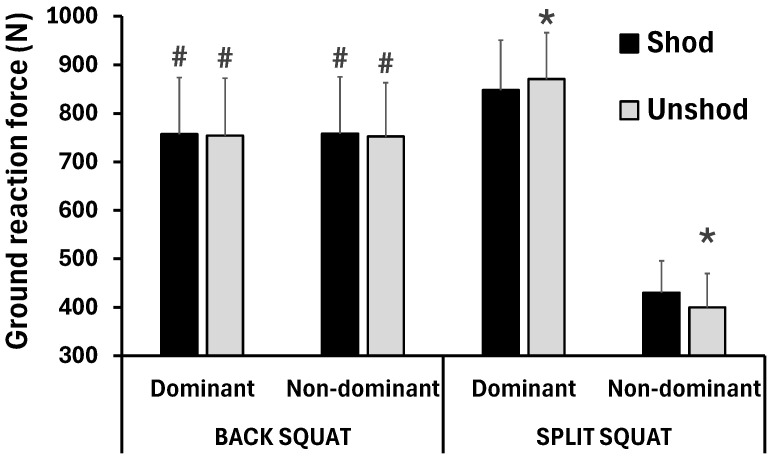
Vertical ground reaction force produced by the dominant (D) and non-dominant (ND) limbs during the back squat and split squat under shod and unshod conditions. * Significant differences (*p* < 0.05) between shod and unshod conditions within the same exercise. # Significant differences (*p* < 0.05) between exercises within the same footwear condition.

Figure 4 shows that, during the back squat, vertical ground reaction force was similarly distributed between the dominant and non-dominant limbs, with no relevant differences between shod and unshod conditions. In contrast, during the split squat, force distribution was clearly asymmetrical (Table 1, see GRF asymmetry), with higher values in the dominant/front limb than in the non-dominant/rear limb. In addition, in this exercise, force produced by the dominant limb was higher under unshod than shod conditions, whereas the non-dominant limb showed the opposite pattern.

## 4. Discussion

The present pilot study examined the effects of conventional sports footwear on ground reaction forces and postural stability during bilateral and unilateral squat-based strength training exercises. Overall, shod conditions were associated with greater CoP displacement and velocity than the unshod conditions, suggesting an acute reduction in postural stability during exercise execution. This effect was exercise-dependent, with larger footwear-related changes during the barbell split squat than during the barbell back squat, particularly in mediolateral CoP variables and inter-limb vertical force distribution. To the authors’ knowledge, this is the first study to examine whether footwear affects postural stability differently during bilateral and unilateral squat-based strength training exercises. Nevertheless, although several of these effects showed large effect sizes, the observed statistical power was not high for all variables, likely due to the small sample size. Therefore, these findings should be interpreted as preliminary and confirmed in future studies including larger samples.

### 4.1. Footwear Effects on Postural Stability

The effect of footwear on postural stability was especially evident in the mediolateral CoP variables. Compared with the unshod condition, the use of footwear increased mediolateral CoP displacement and velocity in both exercises, although the magnitude of this effect was clearly exercise-dependent. In the back squat, the increase was small, ranging from 0.0% to 6.2%, whereas in the split squat, it was much more pronounced, ranging from 18.6% to 28.8%. Similarly, footwear-related differences in CoP sway length were larger in the split squat than in the back squat for both the dominant and non-dominant limbs (Figure 3). These results are consistent with those of Maior and da Silva [23], who observed lower anteroposterior and mediolateral sway after performing the deadlift unshod compared with shod conditions. Although specific evidence on postural stability during strength exercises remains limited, previous studies have shown that footwear can modify the biomechanical execution of exercises such as the back squat or deadlift, affecting movement depth, muscle activation, vertical bar displacement, mechanical work, and joint kinematics [19,20,30]. Therefore, shod or unshod conditions should be considered a relevant methodological and practical factor when analyzing lower-limb strength exercises.

Interestingly, unlike the findings reported by Maior and da Silva [23], the present study showed greater footwear-related differences in the mediolateral than in the anteroposterior direction. This may be explained by the unilateral and asymmetrical nature of the split squat, in which mediolateral balance control is likely to be challenged more than during bilateral exercises. This finding is relevant because previous research has shown that alterations in mediolateral CoP behavior are associated with impaired postural control in different populations, including older adults at risk of falling and individuals with low back pain or ankle instability [31,32]. Therefore, the greater mediolateral CoP displacement and velocity observed under shod conditions, mainly during the split squat, may reflect less efficient control of frontal-plane stability.

### 4.2. Vertical Ground Reaction Force Distribution

Beyond postural stability, the present results also showed that vertical ground reaction force distribution clearly depended on the exercise performed (Table 1 and Figure 4). During the back squat, force was distributed almost symmetrically between the dominant and non-dominant limbs, as expected in a bilateral task. In contrast, during the split squat, force distribution was clearly asymmetrical, with much higher values in the dominant/front limb than in the non-dominant/rear limb under both shod and unshod conditions. This finding is consistent with previous studies showing that split squat variations shift a greater proportion of the mechanical demand toward the front limb. Monajati et al. [33] reported greater quadriceps activation during the split squat compared with the bilateral squat, whereas Knoll et al. [34] described high involvement of different quadriceps portions during unilateral squat tasks. In addition, Kipp et al. [13] showed that the asymmetrical leg position during the split squat increases the demand placed on the front limb, and Schellenberg et al. [35] reported greater forces in the front than in the rear limb during this exercise. Therefore, although the absolute load used in the split squat is lower than in the back squat, the internal force distribution between limbs is much more asymmetrical, concentrating a greater mechanical demand on the front limb.

In addition to the asymmetrical force distribution inherent to the split squat, the present study showed that footwear modified how vertical ground reaction forces were distributed between limbs during this exercise. Specifically, the dominant/front limb produced greater force under unshod than shod conditions, whereas the non-dominant/rear limb showed the opposite pattern (Table 1 and Figure 4). This suggests that footwear may alter the execution strategy of the split squat, redistributing part of the load away from the front limb and toward the rear limb. Previous studies support the idea that small changes in split squat execution can substantially modify load distribution. Escamilla et al. [36] showed that knee loading in the front limb depends on knee angle and step length during lunge-type exercises. Similarly, Schütz et al. [8] reported that load distribution between the front and rear limbs during split squat variations can change by up to 25% depending on tibial inclination and step length. Therefore, the footwear-related changes observed in the present study may be partly explained by subtle modifications in tibial and knee kinematics when the exercise is performed shod compared with unshod. However, this explanation remains speculative because no kinematic data were collected; future studies combining force plate analysis with motion capture would be needed to confirm this mechanism.

However, the small differences observed in total vertical ground reaction force between footwear conditions (Table 1) may be largely attributed to the additional mass of the shoes. Assuming an approximate mass of 300–350 g per shoe, the pair would contribute approximately 6–7 N of additional vertical force, which is of the same order of magnitude as the observed between-condition differences in total vertical ground reaction force. Therefore, after accounting for shoe mass, these differences would likely not remain meaningful and should not be interpreted as evidence of a true increase in force production under shod conditions. It is likely that, if force had been measured directly at the plantar surface of the foot, without including the weight of the footwear, no meaningful differences in force application would have been observed, in line with previous research.

### 4.3. Methodological Considerations and Limitations

Several limitations of the present study should be acknowledged. First, the sample size was small (*n* = 8), and only male participants were included, which may have limited the statistical power of the analysis and the generalizability of the findings, especially to female populations, who may display different postural control strategies. Accordingly, the results should be interpreted as preliminary, particularly regarding interaction effects, and future studies should confirm them in larger and more diverse samples.

Second, although all participants wore conventional running shoes in the shod condition, the shoe model was not standardized. Differences in sole stiffness, cushioning, heel-to-toe drop, or lateral stability may have influenced postural stability and force distribution. Moreover, the unshod condition was performed while participants wore their habitual sports socks and should therefore not be interpreted as a true barefoot condition. Socks may have modified foot–ground friction, plantar sensory feedback, and force transmission compared with direct barefoot contact.

Third, the barbell split squat was used instead of a dumbbell split squat, despite the latter being more common in resistance training practice. However, using a barbell in both exercises reduced potential methodological variability related to load distribution.

Fourth, the familiarization with the posturographic protocol was limited to a single session. Previous studies have suggested that a second familiarization session may substantially improve the reliability of posturographic measurements, mainly by reducing potential learning effects associated with this type of testing [37].

Fifth, the force plates used in the present study recorded only the vertical component of ground reaction force; therefore, inter-limb force asymmetry should be interpreted specifically as inter-limb vertical force asymmetry rather than as a complete three-dimensional loading assessment.

Sixth, although the force plate system used has been validated for vertical force and force–time variables during vertical jump tasks [27,28], its CoP measurements have not, to our knowledge, been independently validated. Nevertheless, CoP location is derived from the vertical forces recorded by four load cells positioned at known locations within each platform, which provides a mechanically plausible basis for the measurement. However, this does not replace specific validation, and the CoP-related findings should therefore be interpreted with caution.

Finally, the present study only examined acute footwear effects during exercise execution; therefore, no conclusions can be drawn regarding chronic adaptations to shod or unshod training.

### 4.4. Practical Implications

From a practical perspective, footwear should be considered when prescribing or assessing squat-based strength exercises, especially unilateral tasks such as the split squat. Conventional sports footwear increased postural sway compared with unshod execution, mainly in the mediolateral direction. Although extrapolation to clinical populations remains speculative, these findings may be particularly relevant in adults or clinical populations with compromised frontal-plane stability, such as older adults with reduced balance capacity or individuals undergoing lower-limb rehabilitation.

Coaches and clinicians should be cautious when using split squat variations to target the front limb, modulate knee loading, or control inter-limb load distribution. When postural stability is a priority, unshod execution or footwear with more stable characteristics may be preferable to conventional running shoes. However, unshod execution should be introduced progressively, since removing footwear may increase mechanical demands on specific foot structures.

## 5. Conclusions

The present study shows that conventional sports footwear should not be considered a neutral condition during squat-based strength training exercises. Compared with unshod execution, footwear was associated with greater postural sway and changes in inter-limb force distribution, particularly during the barbell split squat. These findings suggest that footwear effects become more relevant when the exercise involves an asymmetrical stance and greater mediolateral balance demands. However, these conclusions should be interpreted as preliminary due to the small sample size.

Future studies should include larger samples, participants of both sexes, and standardized footwear models to better characterize the effects of footwear on postural stability and force distribution. In addition, longer familiarization procedures and different split squat variations or loading implements should be considered.

## Figures and Tables

**Table 1:** Descriptive analysis of ground reaction force and center of pressure variables during the back squat and split squat performed under shod and unshod conditions.

	BACK SQUAT		SPLIT SQUAT		Footwear Effect		Exercise Effect		Footwear × Exercise Effect	
Variable	Shod	Unshod	Shod	Unshod	η^2^_p_	Power	η^2^_p_	Power	η^2^_p_	Power
**Total GRF (N)**	1515.1 ± 229.0 *#	1506.1 ± 225.5 #	1278.1 ± 158.2 *	1270.5 ± 158.5	0.910	1.000	0.645	0.862	0.067	0.095
**GRF-D (N)**	756.9 ± 117.0 #	753.9 ± 118.4 #	848.0 ± 102.7 *	870.6 ± 95.2	0.359	0.401	0.611	0.811	0.677	0.906
**GRF-ND (N)**	758.3 ± 116.9 #	752.3 ± 110.8 #	430.1 ± 65.3 *	399.9 ± 69.3	0.646	0.863	0.921	1.000	0.622	0.828
**GRF asymmetry (%)**	5.3 ± 4.5 #	4.6 ± 3.4 #	65.6 ± 9.4 *	74.7 ± 9.0	0.505	0.632	0.981	1.000	0.727	0.957
**ML CoP displacement-D (cm)**	91.4 ± 29.1	87.8 ± 23.2	122.4 ± 42.3	95.0 ± 31.4	0.567	0.738	0.234	0.244	0.536	0.686
**ML CoP displacement-ND (cm)**	88.8 ± 25.6 #	83.6 ± 20.9	123.8 ± 15.9 *	96.4 ± 12.2	0.618	0.822	0.503	0.629	0.550	0.709
**AP CoP displacement-D (cm)**	275.9 ± 70.5 #	258.5 ± 71.6 #	360.5 ± 117.6	327.5 ± 78.9	0.642	0.857	0.742	0.968	0.107	0.125
**AP CoP displacement-ND (cm)**	254.5 ± 28.9 *#	224.3 ± 34.1 #	179.6 ± 49.3	141.9 ± 31.8	0.677	0.905	0.818	0.997	0.016	0.060
**CoP sway length-D (cm)**	312.9 ± 75.8 #	292.3 ± 72.6 #	403.6 ± 127.9	359.9 ± 86.5	0.651	0.870	0.674	0.902	0.204	0.213
**CoP sway length-ND (cm)**	290.9 ± 35.8	258.9 ± 36.4 #	253.6 ± 47.3	199.5 ± 30.3	0.772	0.985	0.608	0.806	0.130	0.144
**ML CoP velocity-D (cm·s^−1^)**	4.9 ± 1.3	4.9 ± 1.2	6.8 ± 1.7	5.7 ± 1.8	0.301	0.324	0.385	0.440	0.444	0.531
**ML CoP velocity-ND (cm·s^−1^)**	4.8 ± 1.2 #	4.7 ± 1.1	7.0 ± 0.8 *	5.9 ± 0.7	0.534	0.682	0.607	0.804	0.582	0.763
**AP CoP velocity-D (cm·s^−1^)**	15.1 ± 3.2 #	14.5 ± 4.3 #	20.2 ± 4.9	19.8 ± 4.2	0.068	0.095	0.861	1.000	0.004	0.052
**AP CoP velocity-ND (cm·s^−1^)**	14.0 ± 1.0 *#	12.6 ± 1.8 #	10.2 ± 2.9	8.6 ± 1.9	0.377	0.427	0.790	0.992	0.004	0.052
**CoP sway velocity-D (cm·s^−1^)**	17.1 ± 3.3 #	16.4 ± 4.3 #	22.6 ± 5.2	21.8 ± 4.7	0.146	0.158	0.831	0.999	0.004	0.053
**CoP sway velocity-ND (cm·s^−1^)**	15.9 ± 1.1 *	14.5 ± 1.8	14.4 ± 2.7	12.1 ± 1.8	0.517	0.652	0.476	0.584	0.088	0.110

Values are expressed as mean ± SD. GRF: ground reaction force; CoP: center of pressure; D: dominant limb; ND: non-dominant limb; ML: mediolateral; AP: anteroposterior. * Significant differences (*p* < 0.05) between shod and unshod conditions within the same exercise. # Significant differences (*p* < 0.05) between exercises within the same footwear condition. Effect sizes (η^2^_p_) were interpreted as trivial (<0.01), small (0.01–0.059), moderate (0.060–0.137), and large (>0.137). Observed statistical power (Power) ranges from 0 to 1, with values ≥0.80 generally considered adequate according to Cohen’s convention [29]. Post hoc comparisons were Bonferroni-adjusted.

## Data Availability

Data are contained within the article or Supplementary Materials.

## Supplementary Materials

The following supporting information can be downloaded at: https://www.mdpi.com/article/10.3390/s26134283/s1, Data S1: Complete dataset containing all individual records included in the study.
